# Biomarker-based risk model to predict persistent multiple organ dysfunctions after congenital heart surgery – A prospective observational cohort study

**DOI:** 10.21203/rs.3.rs-2488327/v1

**Published:** 2023-01-27

**Authors:** Alexis L. Benscoter, Jeffrey A. Alten, Mihir R. Atreya, David S. Cooper, Jonathan W. Byrnes, David P. Nelson, Nicholas J. Ollberding, Hector R. Wong

**Affiliations:** University of Cincinnati College of Medicine, Cincinnati Children’s Hospital Medical Center; University of Cincinnati College of Medicine, Cincinnati Children’s Hospital Medical Center; University of Cincinnati, Cincinnati Children’s Hospital Medical Center; University of Cincinnati College of Medicine, Cincinnati Children’s Hospital Medical Center; University of Alabama at Birmingham; University of Kentucky; University of Cincinnati, Cincinnati Children’s Hospital Medical Center; University of Cincinnati, Cincinnati Children’s Hospital Medical Center

**Keywords:** Cardiopulmonary bypass, inflammation, biomarkers, risk stratification, multiple organ dysfunction, pediatric cardiac critical care

## Abstract

**Background:**

Multiple organ dysfunction syndrome (MODS) is an important cause of post-operative morbidity and mortality for children undergoing cardiac surgery requiring cardiopulmonary bypass (CPB). Dysregulated inflammation is widely regarded as a key contributor to bypass-related MODS pathobiology, with considerable overlap of pathways associated with septic shock. The pediatric sepsis biomarker risk model (PERSEVERE) is comprised of seven protein biomarkers of inflammation, and reliably predicts baseline risk of mortality and organ dysfunction among critically ill children with septic shock. We aimed to determine if PERSEVERE biomarkers and clinical data could be combined to derive a new model to assess the risk of persistent CPB-related MODS in the early post-operative period.

**Methods:**

This study included 306 patients <18 years old admitted to a pediatric cardiac ICU after surgery requiring cardiopulmonary bypass (CPB) for congenital heart disease. Persistent MODS, defined as dysfunction of two or more organ systems on postoperative day 5, was the primary outcome. PERSEVERE biomarkers were collected 4 and 12 hours after CPB. Classification and Regression Tree methodology was used to derive a model to assess the risk of persistent MODS.

**Results:**

The optimal model containing interleukin-8 (IL-8), chemokine ligand 3 (CCL3), and age as predictor variables, had an area under the receiver operating characteristic curve (AUROC) of 0.86 (0.81–0.91) for differentiating those with or without persistent MODS, and a negative predictive value of 99% (95–100). Ten-fold cross-validation of the model yielded a corrected AUROC of 0.75.

**Conclusions:**

We present a novel risk prediction model to assess the risk for development of multiple organ dysfunction after pediatric cardiac surgery requiring CPB. Pending prospective validation, our model may facilitate identification of a high-risk cohort to direct interventions and studies aimed at improving outcomes via mitigation of post-operative organ dysfunction.

**Clinical Trial Registration Number:**

This study does not meet criteria for a clinical trial per the WHO International Clinical Trials Registry Platform as no intervention was performed.

## Background

Cardiopulmonary bypass (CPB) potentiates a systemic inflammatory response in all patients, the degree of which varies based on many factors [1–9]. An exaggerated response, as seen in systemic inflammatory response syndrome (SIRS), can be detrimental and contributes to the development of multiple organ dysfunction (MODS), prolonged length of stay, and worse outcomes [5–7]. Almost all pediatric cardiac surgery patients meet criteria for organ dysfunction in the early postoperative period with ubiquitous inotropic and/or mechanical ventilator support, but children with optimal surgical interventions will begin to wean from postoperative support within the first few days. Failure to wean may represent persistent or progressive organ dysfunction, with risk of mortality increasing in conjunction with number of organ systems involved [10,11]. Identifying patients at increased risk for persistent MODS due to an exaggerated inflammatory response to CPB could help guide clinical management, provide prognostic enrichment in future trials, and, ultimately, improve outcomes.

Sepsis and CPB both cause cellular injury and release of molecules that activate the innate and adaptive immune responses resulting in pro-inflammatory mediator upregulation [1,3]. Research focusing on innate and adaptive immune gene expression and profiling in pediatric sepsis generated the Pediatric Sepsis Biomarker Risk Model (PERSEVERE) [12–20]. PERSEVERE and, more recently, PERSEVERE II, have been utilized as risk-stratification tools to estimate probability of mortality and organ dysfunctions in pediatric septic patients [18]. Research on sepsis and CPB-mediated inflammation has identified significant overlap in inflammatory biomarker activation, including PERSEVERE biomarkers [5,21–27]. We therefore posited that PERSEVERE biomarkers could be used to derive a unique risk model for early prediction of persistent MODS after CPB in pediatric patients.

## Methods

### Patients, samples and data collection

The study was approved by the Institutional Review Board at Cincinnati Children’s Hospital Medical Center. All patients under the age of 18 years old undergoing surgery requiring CPB for correction of congenital heart disease between November 2016 and November 2020 were screened for eligibility. Patients were only included for their index surgery, except patients with single ventricle physiology. Patients with single ventricle physiology could be re-enrolled in the study for each separate surgical stage, i.e., stage 1 palliative surgery, Glenn operation, Fontan operation, and/or biventricular repair. Patients undergoing CPB for heart or lung transplantation, patients requiring immunosuppression, and patients with suspected or proven infection were excluded. Three-hundred and fifty-nine patient encounters (293 unique patients) were consented for the study. Of these, 306 encounters were included in the analysis, because both 4 and 12 hour biomarker samples collected within the specified time. Baseline demographic, clinical, and laboratory data needed to calculate severity of illness scoring and determine organ dysfunction were extracted from the electronic medical record (EMR). To minimize clinically unnecessary blood draws, laboratory data to assess for organ dysfunction was only collected at discretion of the managing clinical team.

### Definitions

The Society of Thoracic Surgery-European Association for Cardiothoracic Surgery (STAT) mortality category [28,29] was used to account for risk related to surgical complexity. Pre- and postoperative severity of illness was assessed using Pediatric Risk of Mortality score III (PRISM III) [30]. Organ dysfunction was defined via adaption of Goldstein criteria [31] to account for differences in the postoperative congenital heart disease population when compared to the pediatric sepsis population, Table 1. Persistent MODS was defined a *priori* as dysfunction of 2 or more organ systems on postoperative day 5. As an additional measure of organ dysfunction, daily Pediatric Logistic Organ Dysfunction-2 [PELOD-2] scores were calculated preoperatively and for the first 5 postoperative days [32,33].

### Clinical and surgical management

All patients received methylprednisolone (30 mg/kg) as part of the CPB circuit prime. Neonates and patients in the hospital prior to their scheduled operation received an additional dose of methylprednisolone (30 mg/kg) the morning of surgery (prior to CPB initiation). Choice of anesthesia was not standardized and left to the decision of the cardiac anesthesiologist. All patients received either modified ultrafiltration and/or continuous ultra ltration intraoperatively, based on surgeon preference. The need for additional steroids and use of postoperative peritoneal dialysis was left to the discretion of the clinical team.

### Biomarker Collection

Biomarkers were collected 4 and 12 hours post-CPB, based on studies suggesting peak inflammation occurs within 24 hours of CPB separation [4–6,8,21,34]. Blood was collected within a +/− 60 minute window, spun down to serum, and stored at −80C until ready to be analyzed. Seven PERSEVERE biomarkers were measured in this study: granzyme B (GZMB), heat shock protein 70 kDa 1B (HSPA1B), interleukin 1a (IL-1a), interleukin 8 (IL-8), C-C chemokine ligand 3 (CCL3), C-C chemokine ligand 4 (CCL4), matrix metalloproteinase 8 (MMP-8). Serum biomarker concentrations were measured according to manufacturer’s instructions using the HSP2MAG-63K multiplex bead platform (MILLIPLEX^™^ MAP Human Sepsis Magnetic Bead Panel 2-Immune Response Multiplex Assay) designed by the EMD Millipore Corporation (Billerica, MA, USA).

### Statistical Analysis

Descriptive statistical analyses were performed using R (version 4.0.4). Demographic, clinical, and biomarker data were described using medians with interquartile ranges (IQR), means with standard deviations, or frequencies with percentages as appropriate. Comparisons of data for patients with and without persistent MODS were performed using the Kruskal-Wallis, chi-squared, or Fisher’s exact tests as appropriate. Multivariate regression analysis, controlling for clinical data, was performed to examine the relationship between biomarker concentrations at 4 and 12 hours and risk for development of MODS.

Classification and regression tree (CART) analysis was used to determine biomarker cut-points and derive a decision tree (Salford Predictive Modeler v6.6, Salford Systems, San Diego, CA) [35]. Candidate prediction variables for derivation of the decision tree were as follows: all seven PERSEVERE biomarkers at 4 and 12 hour time points, change in PERSEVERE biomarker levels from 4 to 12 hours, age in months (included as both a continuous and dichotomous variable), single ventricle status, history of prematurity, CPB time, maximum vasoactive inotropic score (VIS) and STS-EACTS mortality category. Clinical predictor variable selection was based on extant literature [36–39]. Tuning parameters determined a priori included: 10-fold cross validation, at least one of the paired terminal daughter nodes contains ≥ 5% of the subjects in the root node, and no predictor variables repeated within one of the two main branches. Performance of the decision tree was determined by generating a Classification table of true versus predicted status and calculation of discrimination metrics including sensitivity, specificity, positive and negative predictive values, and area under the receiver operating curve (AUROC). We compared our prediction model, which we will refer to as PERSEVERE-CPB, to PRISM III and STS-EACTS mortality category, as they are widely accepted and validated risk assessment and severity of illness scoring systems this patient population, using the AUROC, sensitivity, and specificity. We further compared PERSEVERE-CPB to the 24 hour postoperative PELOD-2 score, as PELOD-2 is a validated scoring system for organ dysfunction [32].

IL-8 was the first level decision rule in PERSEVERE-CPB. As such, we generated a Classification table to evaluate the utility of IL-8 as an independent predictor of MODS.

Finally, we stratified the cohort into risk category based on high, intermediate, and low risk terminal nodes of our model. Using risk categories (referred to as PERSEVERE risk category), we evaluated the association of risk and administration of post-operative steroids for hypotension.

## Results

Demographics, clinical characteristic, and biomarker concentrations of patients with and without persistent MODS in Table 2 and Table 3. Of the 306 subjects with biomarkers drawn at both 4 and 12 hours after separation from CPB, 43 (14.1%) had persistent MODS on POD 5. The cohort with persistent MODS was significantly younger, had a history of prematurity, had higher illness severity before and immediately after CPB, received more organ support, were more likely to receive steroids for post-operative hypotension, and had worse clinical outcomes. When controlling for age less than 12 months, STAT, CPB time, and single ventricle status, IL-8 concentration at both 4 and 12 hours correlated with the development of persistent MODS, as did 12 hour concentrations of GZMB and CCL3, Table 4.

### Biomarker-based Risk Prediction Model

**Figure 1:**
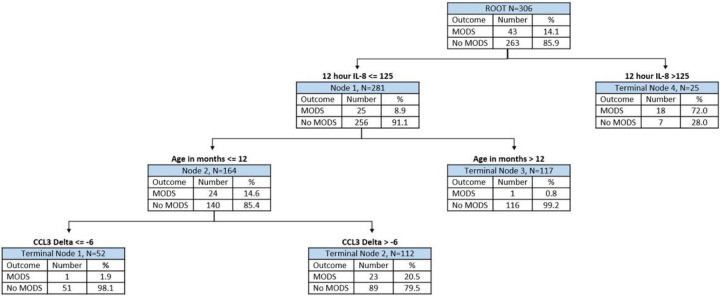
Derivation classification tree for PERSEVERE-CPB model The Classification tree consists of two biomarker-based decision rules and one clinically based decision rule. The 12-hour interleukin-8 (IL8) serum concentration and the change in C-C chemokine ligand 3 (CCL3) serum concentration from 4 to 12 hours were included. Each node contains the total number of subjects meeting the biomarker concentration or clinically based decision rule criteria, the number of subjects with or without persistent multiple organ dysfunction syndrome (MODS) at postoperative day (POD) 5, and the percentage of each respective outcome. Terminal nodes 1 and 3 were considered low-risk nodes, with subjects being less likely to develop persistent MODS. Terminal nodes 2 and 4 were considered high-risk and more predictive of development of persistent MODS. The area under the curve (AUC) for this tree was 0.86, with cross-validated estimate for AUROC of 0.75.

Our newly derived PERSEVERE-CPB model is shown in Figure 1. PERSEVERE-CPB included IL-8 concentration at 12 hours, the change in serum concentration of CCL3 from 4 to 12 hours, and infant age category (< 12 months). There were two low-risk terminal nodes (terminal nodes 1 and 3) in which subjects had <2% risk of developing persistent MODS. There was one intermediate-risk node with 23 patients (20.5%) who developed persistent organ dysfunction (terminal node 2). There was one high-risk node with persistent organ dysfunction in 72% of patients (terminal node 4). PERSEVERE-CPB performed well at determining risk of persistent MODS with model characteristics shown in Table 5. Although there were multiple different univariate associations with MODS (Table 2), the addition of these candidate variables (single ventricle status, STAT, history of prematurity, CPB time, and maximum VIS) did not improve the performance of PERSEVERE-CPB (data not shown).

### Prediction performance

**Figure 2:**
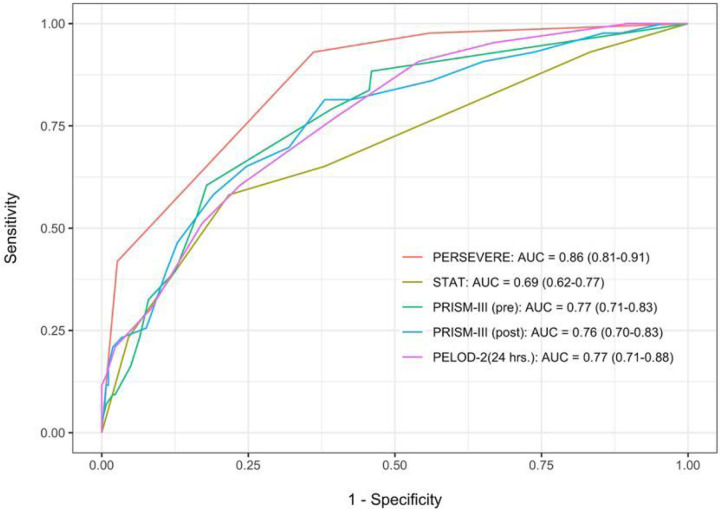
Comparison of PERSEVERE-CPB to validated risk-assessment tools to predict persistent MODS. PERSEVERE-CPB functioned well as a predictor of multiple organ dysfunction syndrome, with cross-validation area under the curve (AUC) that was comparable to validated risk-assessment tools in our cohort. PERSEVERE: PERSEVERE-CPB biomarker prediction model, STAT: Society of Thoracic Surgery-European Association for Cardiothoracic Surgery mortality category, PRISM-III (pre): Pediatric Risk of Mortality score calculated using preoperative data, PRISM-III (post): Pediatric Risk of Mortality score calculated using data from the first 24 hours after surgery, PELOD-2: Pediatric Logistic Organ Dysfunction Score-2

PERSEVERE-CPB had excellent performance for prediction of MODS: AUROC, 0.86 (95% CI 0.81, 0.91), Figure 2. After cross validation, our model’s corrected AUROC (0.75) still had good performance. PERSEVERE-CPB performed favorably to other validated risk scoring systems for prediction of MODS in our study cohort: STAT, 0.69 (0.62, 0.77), preoperative PRISM III, 0.77 (0.71, 0.83), and postoperative PRISM III, 0.76 (0.70, 0.83). PELOD-2 calculated using data from the first 24 hours after CPB had an AUROC of 0.77 (0.71, 0.88).

### IL-8 as a predictor of MODS

IL-8 predicted risk of MODS fairly well in our cohort: AUROC, 0.74 (0.66, 0.81) and 0.77 (0.70, 0.84) using 4 and 12 hour levels, respectively. At both time points, IL-8 had high negative predictive values (NPV) and low negative likelihood ratios (−LR): NPV 92% (86%, 94%) and –LR 0.53 (0.37, 0.74) using 4 hour levels, NPV 94% (88%, 95%) and –LR 0.42 (0.27, 0.64) using 12 hour levels, **Additional File 1**.

### Assessment of Steroid Need and Outcome by Risk Category

The portion of the cohort falling into the high-risk PERSEVERE-CPB category (terminal node 4 of model) were more likely to receive steroids for post-operative hypotension compared to those falling into the intermediate- and low-risk categories (35%, 22%, 2%, respectively, p<0.001), **Additional File 2.**

## Discussion

Using inflammatory biomarkers and established clinical risk factors, we have derived a decision tree that is able to predict patients at greatest risk for persistent multiple organ dysfunction syndrome at post-operative day 5 after cardiopulmonary bypass surgery for congenital heart disease. Similar to previous studies, this study shows that interleukin-8 (IL-8) likely plays a significant role in early inflammation related postoperative morbidity.

With high sensitivity, PERSEVERE-CPB identifies patients most at risk for persistent MODS, and its associated morbidity and mortality. As illustrated by a high negative predictive value and low negative likelihood ratio, our model may serve as an early postoperative screening tool for persistent MODS, since it correctly identified nearly all patients who develop persistent MODS (99.9% based on true positive and false negative data). This could potentially facilitate risk stratification, prognostication, and targeted clinical interventions as early as 12 hours after congenital heart surgery. Additionally, early identification of a high risk cohort may aid new therapies aimed at reducing risk of MODS, which may guide patient selection for future clinical trials. However, given the low positive predictive value and low positive likelihood ratio, PERSEVERE-CPB over-selects for MODS (approximately 31% false positive rate), which may limit its utility. In future work, addition of real-time physiologic and laboratory data to the model may improve the precision and specificity of this model.

For assessing risk of persistent MODS, PERSEVERE-CPB performed well when compared to existing pediatric critical care and cardiac surgery risk-assessment tools (STAT, PRISM III, PELOD 2). In particular, PERSEVERE-CPB performed similarly to the post-operative day one PELOD-2 score for predicting development of persistent MODS. Although STAT and PRISM III were primarily validated to predict risk of mortality and not MODS, the low mortality rate in our cohort did not allow us to develop a biomarker-based predictive model for in-hospital mortality

IL-8 level functioned as the upper level decision rule, indicating that it played a key role in determination of risk for MODS. Almost 42% of patients who developed persistent MODS fell into terminal node 4, with an elevated 12 hour IL-8 concentration. IL-8 is one of the more studied biomarkers of inflammation in patients after CPB. It plays a pivotal role in neutrophil activation and is produced in large quantities by endothelial cells [40]. Elevated postoperative IL-8 has been associated with markers of low cardiac output (low mixed venous oxygen concentration and higher inotropic score) [41], development of postoperative acute kidney injury [26,42,43], increased duration of mechanical ventilation [22,43], and longer ICU length of stay [6]. The pathophysiologic role IL-8 plays in neutrophil/endothelium activation, bypass-mediated inflammation, and development of MODS warrants further examination, with obvious potential as a therapeutic target.

Unlike IL-8, CCL3, or macrophage inflammatory protein 1a (MIP-1a), has not been extensively studied in bypass-mediated inflammation. During acute inflammation, CCL3 aids in the recruitment of leukocytes and plays a role in neutrophil infiltration [45,46]. Since both PERSEVERE and PERSEVERE-II have demonstrated CCL3 plays a major role in discrimination of both mortality and multiple organ failure in severe pediatric sepsis [47], further investigation into the role of CCL3 in CPB-mediated inflammation and its contribution to development of organ dysfunction is warranted.

Although use of systemic steroids in our patients may have blunted the bypass-mediated inflammatory response, we were unable to assess this in our study since all patients received steroids. When stratified by risk category, the high risk cohort was more likely to receive steroids for hypotension in the first 24 hours postoperative, which may reflect an enhanced inflammatory response leading to higher degree or longer lasting vasoplegia (**Additional File 2**).

Non-uniform perioperative steroid administration in our population is a limitation of this study. All preoperatively hospitalized neonates and infants received steroids both before and during CPB, whereas all other patients received steroids only during CPB. The effects of additional steroids on biomarker levels in our study population is unknown. Another limitation of the study was placement of peritoneal dialysis catheters in neonates, which is a standard practice at our institution. Use of peritoneal dialysis has been shown to decrease inflammatory cytokines after bypass and in other inflammatory states [48]. Dialysis was used in 12 patients in the first 24 hours after separation from CPB, which likely contributed to lower biomarker concentrations. Consistent with this supposition, IL-8 and CCL-3 concentrations were significantly lower in patients who received dialysis (data not shown). Peritoneal dialysis catheters drained ascites without active dialysis in the remaining 34 neonates. Also limiting our study, 3 patients who developed MODS had a residual lesion or complication of care that contributed to prolonged need for mechanical ventilation and inotropic/vasopressor support. It would have been preferable to include only patients with MODS resulting from biologic and physiologic consequences of surgery and their intrinsic response to inflammation. Lastly, although the small number of events in this study prevented validation beyond a 10-fold cross validation procedure, we hope to be able to enhance this in a future, multicenter study. Cross-validation AUC for our model showed acceptable ability to predict persistent MODS, comparable to postoperative PRISM III and PELOD-2.

## Conclusions

Using known clinical risk factors and biomarkers of inflammation originally identified as key markers of inflammation in pediatric patients with septic shock, we have created a simple, biologically plausible model that accurately predicts risk of persistent organ dysfunction in pediatric patients after cardiac surgery for congenital heart disease. IL-8 appears to contribute to the development of MODS after CPB in our patient population, future efforts to better define CPB related IL-8 pathophysiology and modifiable risk factors for IL-8 elevation after CPB are warranted.

## Figures and Tables

**Table 1:** Definitions of Organ Dysfunction

Organ System	Definition of Dysfunction
Cardiovascular	On vasoactive drugs by POD 5
	or persistent lactatemia >5 mmol/L
	or hypotension < 5^th^ percentile for age or systolic blood pressure < 2 SD below normal for age
Respiratory	Need for invasive or non-invasive ventilation by POD 5
	or persistent respiratory acidosis with PaCO2 > 65 mmHg or 20 mmHg above baseline
	or PaO2/FiO2 <300 torr in absence of cyanotic heart disease or preexisting lung disease
Renal	Cr >2 times upper limit of normal for age or 2-fold increase in baseline Cr, need for dialysis
GI/Hepatic	Total bilirubin > 4 mg/dL (outside of newborn period) or ALT 2 times upper normal limit for age, development of NEC
Hematologic	Platelet count < 80,000/mm^3^
	or INR > 2 in a patient not on warfarin
Neurologic	GCS < 11 in a non-sedated patient
	or acute mental status change with decrease in GCS of > 3 from baseline
	or new cerebrovascular accident

POD: post-operative day; SD: standard deviation; PaCO2: arterial partial pressure of carbon dioxide; PaO2: arterial partial pressure of oxygen; FiO2: fraction of inspired oxygen; Cr: creatinine; ALT: alanine transaminase; NEC: necrotizing enterocolitis; INR: international normalized ratio; GCS: Glascow coma score

**Table 2:** Demographics and clinical characteristics

	All*	MODS*	No MODS*	p value
**Number of subjects (%)**	306	43 (14.0)	263 (86.0)	-
**Age (months**	6 (3–42.9)	2 (0.2–5.3)	8 (3.9–48)	<0.001
**Number of females (%)**	134 (43.8)	20 (46.5)	114 (43.3)	0.70
**Race, number (%)**				0.31
**White, non-Hispanic**	269 (87.9)	34 (79.1)	235 (89.3)	
**White, Hispanic**	6 (2.0)	2 (4.7)	4 (1.5)	
**Black**	23 (7.5)	6 (13.9)	17 (6.5)	
**Other**	8 (2.6)	1 (2.3)	7 (2.7)	
**Number of neonates (%)**	43 (14.1)	17 (39.5)	26 (9.9)	<0.001
**Number of single ventricle patients (%)**	117 (38.2)	23 (53.5)	94 (35.7)	0.026
**Number of infants (%)**	182 (59.5)	38 (88.4)	144 (54.8)	<0.001
**Number of infants born premature (%)**	45 (14.7)	13 (30.2)	32 (12.2)	0.002
**STAT, number (%)**				<0.001
**1**	47 (15.4%)	3 (7.0)	44 (16.7)	
**2**	131 (42.8%)	12 (27.9)	119 (45.2)	
**3**	46 (15.2%)	3 (7.0)	43 (16.3)	
**4**	60 (19.6%)	15 (34.9)	45 (17.1)	
**5**	22 (7.2%)	10 (23.2)	12 (4.6)	
**CPB time in minutes**	138.0 (92.3; 183.0)	176.0 (112.0; 206.5)	132.0 (89.0; 179.0)	0.005
**Number receiving MUF (%)**	195 (63.7)	30 (69.8)	165 (62.7)	0.374
**Pre-op PRISM III**	2.0 (0.0; 3.0)	5.0 (3.0; 7.0)	0.0 (0.0; 3.0)	<0.001
**Post-op PRISM III**	8.0 (6.0; 12.0)	13.0 (10.0; 16.0)	8.0 (5.0; 11.0)	<0.001
**PELOD-2 preoperative**	0.0 (0.0; 2.0)	2.0 (0.0; 2.0)	0.0 (0.0; 2.0)	<0.001
**PELOD-2 24 hours postoperative**	4.0 (2.0; 6.0)	7.0 (5.0; 8.0)	4.0 (2.0; 5.0)	<0.001
**VIS at 4 hours post-CPB**	7.0 (5.0; 10.0)	8.0 (7.0; 11.8)	7.0 (4.5; 9.0)	0.007
**Maximum VIS**	7.0 (5.0; 15.4)	17.5 (14.5; 26.0)	7.0 (5.0; 12.5)	<0.001
**Lowest pH**	7.29 (7.26–7.33)	7.25 (7.2; 7.3)	7.3 (7.3; 7.3)	<0.001
**Peak lactate**	2.4 (1.6–4.0)	3.7 (2.3; 6.3)	2.2 (1.5; 3.8)	<0.001
**Number receiving steroids postoperative (%)**	70 (22.9)	24 (55.8)	46 (17.5)	<0.001
**Number receiving steroids for hypotension postoperative (%)**	27 (8.8)	13 (30.2)	14 (5.3)	<0.001
**Ventilator-free days**	27.0 (26.0; 28.0)	17.0 (13.0; 23.0)	28.0 (26.0; 28.0)	<0.001
**Vasoactive-free days**	26.0 (25.0; 27.0)	20.0 (14.5; 22.0)	27.0 (26.0; 27.0)	<0.001
**Number of in-hospital mortality (%)**	7 (2.3)	6 (14.0)	1 (0.4)	<0.001
**Number alive and out of the hospital by POD 28 (%)**	267 (87.3)	20 (46.5)	247 (93.9)	<0.001
**CICU LOS**	3.0 (2.0; 8.0)	15.0 (11.0; 34.0)	3.0 (2.0; 4.5)	<0.001
**Hospital LOS**	7.0 (4.0; 15.0)	24.0 (19.0; 67.0)	7.0 (4.0; 11.0)	<0.001

All data is presented as median (interquartile range) unless specified; MODS: persistent multiple organ dysfunction at postoperative day 5; neonate: <30 days old; infant: <12 month old; STAT: Society of Thoracic Surgery-European Association for Cardiothoracic Surgery mortality category; CPB: cardiopulmonary bypass; PRISM: Pediatric Risk of Mortality score; PELOD-2: Pediatric Logistic Organ Dysfunction Score-2; VIS: vasoactive inotropic score; POD: postoperative day; CICU: cardiac intensive care unit; LOS: length of stay

**Table 3:** Comparison of biomarker concentrations in patients with and without persistent MODS

	All	MODS	No MODS	p-value
**GZMB (pg/mL)**				
4 hour	5.8 (2.5; 14.5)	5.5 (2.4; 20.3)	5.8 (2.6; 13.9)	0.51
12 hour	1.5 (0.3; 3.1)	2.0 (0.4; 3.3)	1.5 (0.3; 3.0)	0.28
**HSP70 (pg/mL)**				
4 hour	44.4 (31.2; 63.4) ×10^4^	52.8 (32.8; 75.6) ×10^4^	43.9 (31.0; 61.6) ×10^4^	0.93
12 hour	23.1 (14.3; 35.6) ×10^4^	32.1 (17.8; 51.9×10^5^)	22.6 (13.8; 32.9) ×10^4^	0.35
**IL-1 a (pg/mL)**				
4 hour	0.1 (0.01; 0.6)	0.01 (0.0; 0.6)	0.01 (0.0–0.6)	0.81
12 hour	0.1 (0.0; 0.6)	0.01 (0.0; 0.6)	0.01 (0.0–0.6)	0.48
**IL-8 (pg/mL)**				
4 hour	51.5 (24.9; 97.9)	139.6 (46.1; 342.5)	45.7 (22.3; 86.8)	<0.001
12 hour	37.4 (16.5–69.2)	90.4 (40.9; 210.0)	32.7 (14.5; 55.5)	<0.001
**CCL3 (pg/mL)**				
4 hour	23.4 (13.3; 41.9)	25.5 (13.5; 44.1)	22.9 (13.1; 41.9)	0.42
12 hour	22.5 (13.1; 22.5)	31.4 (18.2; 52.4)	21.1 (11.9; 35.0)	<0.001
**CCL4 (pg/mL)**				
4 hour	56.5 (35.3; 84.3)	53.8 (34.5; 92.4)	56.5 (35.7; 82.7)	0.06
12 hour	35.6 (24.3; 61.0)	49.7 (24.4; 69.5)	38.2 (24.3; 58.8)	0.05
**MMP-8 (pa/mL)**				
4 hour	1915.6 (975.7; 4307.1)	1976.6 (932.1; 5221.5)	1912.5 (1002.7; 4248.9)	0.67
12 hour	1519.1 (722.6; 3687.4)	1450.4 (717.9; 4484.2)	1526.3 (720.0; 3574.5)	0.47

Data is presented as median (interquartile range); MODS: persistent multiple organ dysfunction at postoperative day 5; GZMB, granzyme B; HSPA1B, heat shock protein 70 kDa 1B; IL-1a interleukin 1a; IL-8, interleukin 8; CCL3, C-C chemokine ligand 3; CCL4, C-C chemokine ligand 4; MMP-8, matrix metalloproteinase 8

**Table 4:** Development of MODS based on PERSEVERE biomarkers

Biomarkers at 4 hours		
	OR (95% CI)	p-value
**GZMB**	0.79 (0.20; 1.18)	0.524
**HSP70**	0.94 (0.35; 1.35)	0.852
**IL-1a**	1.08 (0.50; 1.45)	0.684
**IL-8**	1.94 (1.41; 2.77)	<0.001
**CCL3**	1.07 (0.74; 1.49)	0.700
**CCL4**	1.21 (0.88; 1.64)	0.210
**MMP-8**	1.15 (0.69; 1.60)	0.473
Biomarkers at 12 hours		
	OR (95% CI)	p-value
**GZMB**	1.42 (1.04; 1.88)	0.012
**HSP70**	1.27 (0.89; 1.65)	0.081
**IL-1a**	0.69 (0.18; 1.23)	0.394
**IL-8**	11.42 (2.91; 57.11)	0.001
**CCL3**	1.36 (1.02; 1.84)	0.038
**CCL4**	1.27 (0.92; 1.71)	0.125
**MMP-8**	0.99 (0.46; 1.44)	0.963

Odds ratios (OR) obtained via logistic regression. Each biomarker was modeled separately. All models adjusted for age less than 12 months (infant), STAT mortality category, single ventricle status, and time (in minutes) on cardiopulmonary bypass. CI: confidence interval; ; GZMB, granzyme B; HSPA1B, heat shock protein 70 kDa 1B; IL-1a interleukin 1a; IL-8, interleukin 8; CCL3, C-C chemokine ligand 3; CCL4, C-C chemokine ligand 4; MMP-8, matrix metalloproteinase 8

**Table 5:** Diagnostic test characteristics of PERSEVERE-CPB

**Number of subjects**	306
**Number of True Positives**	41
**Number of True Negatives**	167
**Number of False Positives**	96
**Number of False Negatives**	2
**Sensitivity**	95% (83; 99)
**Specificity**	64% (57; 69)
**Positive Predictive Value**	30% (23; 38)
**Negative Predictive Value**	99% (95; 100)
**+Likelihood Ratio**	2.6 (2.2; 3.1)
**−Likelihood Ratio**	0.07 (0.02; 0.28)
**AUC**	0.86 (0.81;0.91)
**Cross Validation AUC**	0.75

Numbers in parenthesis represent 95% confidence intervals. AUC: area under the curve; + likelihood ratio: positive likelihood ratio; - likelihood ratio: negative likelihood ratio

## Data Availability

The datasets used and/or analyzed during this study are available from the corresponding author on reasonable request.

## Abbreviations

MODS: multiple organ dysfunction syndrome
CPB: cardiopulmonary bypass
SIRS: systemic inflammatory response syndrome
PERSEVERE: Pediatric Sepsis Biomarker Risk Model
POD: postoperative day
PaCO2: arterial partial pressure of carbon dioxide
PaO2: arterial partial pressure of oxygen
FiO2: fraction of inspired oxygen
Cr: creatinine
ALT: alanine transaminase
NEC: necrotizing enterocolitis
INR: international normalized ratio
GCS: Glascow coma score
CICU: cardiac intensive care unit
STAT: Society of Thoracic Surgery-European Association for Cardiothoracic Surgery mortality category
PRISM III: Pediatric Risk of Mortality score III
PELOD-2: Pediatric Logistic Organ Dysfunction Score-2
VIS: vasoactive inotropic score
LOS: length of stay
GZMB: granzyme B
HSPA1B: heat shock protein 70 kDa 1B
IL-1a: interleukin 1a
IL-8: interleukin 8
CCL3: C-C chemokine ligand 3
CCL4: C-C chemokine ligand 4
MMP-8: matrix metalloproteinase 8
